# High Fat Diet Enhances β-Site Cleavage of Amyloid Precursor Protein (APP) via Promoting β-Site APP Cleaving Enzyme 1/Adaptor Protein 2/Clathrin Complex Formation

**DOI:** 10.1371/journal.pone.0131199

**Published:** 2015-09-28

**Authors:** Masato Maesako, Maiko Uemura, Yoshitaka Tashiro, Kazuki Sasaki, Kiwamu Watanabe, Yasuha Noda, Karin Ueda, Megumi Asada-Utsugi, Masakazu Kubota, Katsuya Okawa, Masafumi Ihara, Shun Shimohama, Kengo Uemura, Ayae Kinoshita

**Affiliations:** 1 Department of Human Health Sciences, Kyoto University Graduate School of Medicine, Kyoto, Japan; 2 Department of Neurology, Kyoto University Graduate School of Medicine, Kyoto, Japan; 3 SK project Medical Innovation Center, Kyoto University Graduate School of Medicine, Kyoto, Japan; 4 Kyowa Hakko Kirin Co., Ltd, Shizuoka, Japan; 5 Department of Neurology, National Cerebral and Cardiovascular Center, Osaka, Japan; 6 Department of Neurology, Sapporo Medical University, Sapporo, Japan; 7 Ishiki Hospital, Kagoshima, Japan; National Center for Geriatrics and Gerontology, JAPAN

## Abstract

Obesity and type 2 diabetes are risk factors of Alzheimer’s disease (AD). We reported that a high fat diet (HFD) promotes amyloid precursor protein (APP) cleavage by β-site APP cleaving enzyme 1 (BACE1) without increasing BACE1 levels in APP transgenic mice. However, the detailed mechanism had remained unclear. Here we demonstrate that HFD promotes BACE1/Adaptor protein-2 (AP-2)/clathrin complex formation by increasing AP-2 levels in APP transgenic mice. In Swedish APP overexpressing Chinese hamster ovary (CHO) cells as well as in SH-SY5Y cells, overexpression of AP-2 promoted the formation of BACE1/AP-2/clathrin complex, increasing the level of the soluble form of APP β (sAPPβ). On the other hand, mutant D495R BACE1, which inhibits formation of this trimeric complex, was shown to decrease the level of sAPPβ. Overexpression of AP-2 promoted the internalization of BACE1 from the cell surface, thus reducing the cell surface BACE1 level. As such, we concluded that HFD may induce the formation of the BACE1/AP-2/clathrin complex, which is followed by its transport of BACE1 from the cell surface to the intracellular compartments. These events might be associated with the enhancement of β-site cleavage of APP in APP transgenic mice. Here we present evidence that HFD, by regulation of subcellular trafficking of BACE1, promotes APP cleavage.

## Introduction

Characteristic of Alzheimer’s disease (AD) pathology is formation of the amyloid plaque. The amyloid plaque is composed of β-amyloid (Aβ), a peptide fragment of the amyloid precursor protein (APP) produced when cleaved in sequence by β- and γ-secretase. β-secretase cleaves APP at the extramembrane domain, producing the soluble form of APP β (sAPPβ) and the APP C-terminus fragment β (APP-CTFβ). γ-Secretase subsequently cleaves APP-CTFβ at the intramembrane domain, producing Aβ and the APP intracellular domain (AICD). Many familial AD mutations have been located on the gene responsible for APP [1–3], and by taking advantage of these pathogenic mutations, many strains of APP transgenic mice have been established for use as AD model mice [4–6]. β-site APP cleaving enzyme 1 (BACE1) is classified as a β-secretase [7, 8] and a significant increase in BACE1 activity has been reported in sporadic AD brains [9]. BACE1 activity is regulated by its subcellular trafficking and/or interacting proteins (reviewed in [10, 11]). The first step in Aβ production is the cleavage of APP by BACE1, thus making BACE1 a therapeutic and/or preventive target in AD research.

Obesity and type 2 diabetes are known to be risk factors of AD [12–14]. An epidemiological study suggested that individuals following diets with high caloric intake have a 1.5 times greater risk of AD than those with low caloric intake [15]. Moreover, many reports have shown that the application of high fat diet (HFD) in APP transgenic mice increases Aβ deposition [16–20]. Some reports demonstrated that HFD inhibits Aβ degradation and/or clearance [17, 18], while others argued that HFD promotes Aβ production [16, 20]. Drawing on a proposed mechanism for the latter, some groups suggested that HFD increases the expression level of BACE1 [21–23]. However, we have previously reported that HFD increases the level of APP-CTFβ without changing APP or BACE1 levels, indicating that HFD may strengthen the activity of BACE1, followed by promotion of the cleavage of APP [20], rather than increasing the BACE1 protein level. How HFD promotes the cleavage of APP by BACE1 has remained unclear until now.

Here we demonstrate that HFD promoted the formation of BACE1/Adaptor protein-2 (AP-2)/clathrin complex by increasing AP-2 levels in APP transgenic mice. In Swedish APP overexpressing Chinese hamster ovary (CHO) cells as well as in SH-SY5Y cells, promotion of BACE1/AP-2/clathrin complex formation by AP-2 overexpression increased the level of sAPPβ. Conversely, disruption of the complex using an artificial BACE1 mutation (D495R BACE1) decreased the level of sAPPβ. Overexpression of AP-2 promoted the internalization of BACE1 from the cell surface, and it reduced the level of cell surface BACE1. Moreover, D495R BACE1 mainly localized at the cell surface but wild type (WT) BACE1 did within clathrin vesicles. Therefore, we concluded that HFD might induce the formation of BACE1/AP-2/clathrin complex, thus transporting BACE1 from the cell surface to the intracellular compartments. Our results show that this leads to enhancement of the β-site cleavage of APP in APP transgenic mice. These results present strong evidence that HFD, by the regulation of subcellular trafficking of BACE1, may promote its cleavage of APP.

## Material and Methods

### Ethics statement

All animal experiments in this study were performed with the approval of the Animal Experiment Committees of Kyoto University, Graduate School of Medicine (Permit Number: 14521). All experiments were in strict accordance with the relevant international guidelines and all efforts were made to minimize suffering.

### Animals and dietary condition

APP transgenic mice overexpressing familial mutations bearing both Swedish (K670N/M671L) and Indiana (V717F) mutation were imported from the Jackson Laboratory (USA). They were maintained as heterozygotes. Age-matched APP transgenic mice were either exposed to standard diet (10% fat, 70% carbohydrate, and 20% protein, Oriental Yeast Co., Ltd., Japan) or to HFD (caloric composition, 60% fat, 20% carbohydrate, and 20% protein, Research Diet, Inc., Canada) for 20 weeks (5 months), from 2–3 to 7–8 months of age. After dietary manipulation, mice were deeply anesthetized by tribromoethanol, and transcardially fixed by 4% paraformaldehyde, followed by perfusion with PBS, and then brains were extracted. Extracted brains were cut sagittally into left and right hemispheres. The left hemisphere was re-fixed in 4% paraformaldehyde for histological analysis. After removing the olfactory lobe and the cerebellum, the right hemisphere was rapidly frozen in liquid nitrogen for biochemical analysis.

### Cell culture and transfection

CHO cells stably overexpressing Swedish mutant APP (K670M/ N770L) (APPsw/Ncad-CHO cells), in which it is possible to quantify released Aβ, were obtained [24] and maintained as described previously [25, 26]. SH-SY5Y cells which derived from human neuroblastoma were maintained in Opti-MEM (Life technology, USA) containing 10% FBS. For transient transfection into the CHO cells as well as into SH-SY5Y cells, we used TransFectin reagent (Bio-Rad, USA) according to the manufacturer’s protocol. Reporter gene activity was assayed from 24 to 48 hours after transfection.

### Immunoprecipitation and proteomics

The cerebrum of our mice was extracted in radio-immunoprecipitation assay (RIPA) buffer (50 mM Tris-HCl, 150 mM NaCl, 1% Triton X100, 1% NP-40, 0.5% Deoxycholate, 0.1% SDS, pH 8.0) (40 mg/500 μl) with protease inhibitor cocktail (Roche Diagnostic, Germany) and sufficiently homogenized on ice. To conduct an immnoprecipitation assay using APPsw/Ncad-CHO cells, the cells were cultured in 6cm dishes at a density of 2.4 × 10^6^ cells/dish and 48 hours after incubation, the cells were transfected with the constructs. Then, the cells were washed twice in PBS 24 hours after transfection, and scraped off. The cell pellets were suspended in TNE buffer (10 mM Tris HCl, 150 mM NaCl, 1% NP-40, 1 mM EDTA, pH 7.8) supplemented with protease inhibitor cocktail. Each sample was then centrifuged at 14,000 g for 20 minutes at 4°C, and the supernatants were collected. Protein concentrations were determined using the Bradford assay [27]. For the proteomic analysis, equal amounts of aliquots were treated with 20 μl protein G-Sepharose (GE Healthcare, Sweden) for 1 hour at 4°C. After removing protein G-Sepharose by centrifugation at 14,000 g for 20 minutes, 3 μg of monoclonal anti-BACE1 antibody (Chemicon, USA) was added to the supernatants. Each sample was rotated for 1 hour at 4°C, and then treated with 40 μl protein G-Sepharose for 1 hour at 4°C. The immunoprecipitates were washed with RIPA buffer 4 times, and resuspended in 20 μl 2 × sample buffer (125 mM Tris-HCl, 4% SDS, 20% glycerol, 10% 2-mercaptoethanol, and 0.01% bromophenol blue, pH 6.8). After boiling for 5 minutes and centrifuging at 14,000 g for 5 minutes, the supernatants were subjected to SDS-PAGE. To visualize proteins, the gels were stained with silver nitrate using PlusOne Silver Staining Kit, Protein (GE Healthcare). The protein bands were excised and subjected to in gel trypsinization. Molecular mass analysis of the tryptic peptides was performed by MALDI-MS with an ultrafleXtreme (Bruker Daltonics, Germany).

### Antibodies

Mouse monoclonal anti-BACE1 antibody was from Chemicon. Rabbit polyclonal anti-BACE1 antibody was from Calbiochem (USA). Mouse monoclonal anti-clathrin heavy chain and rabbit polyclonal anti-Golgi-localized, gamma adaptin ear-containing, ARF-binding protein 1 (GGA1), goat polyclonal anti-HA tag antibodies were from Abcam (UK). Mouse monoclonal anti-Adaptin α antibody recognizing AP-2 α-subunit was from BD Transduction Laboratories (USA). Mouse monoclonal anti-β-actin, rabbit polyclonal anti-APP C-terminus, mouse monoclonal anti-Aβ 6E10, rabbit polyclonal anti-HA tag antibodies were from SIGMA (USA). Rabbit polyclonal anti-myc tag antibody was from Millipore (USA). Mouse monoclonal anti-myc tag antibody was from Cell Signaling Technology (USA). Mouse monoclonal anti-transferrin receptor (TfR) antibody was from Invitrogen (USA). Goat polyclonal anti-Early Endosome Antigen 1 (EEA1), goat polyclonal anti-Lysosome-associated membrane protein 2 (Lamp2), normal anti-mouse IgG and normal anti-rabbit IgG were from Santa Cruz Biotechnology, Inc (USA). Alexa Fluor 546 mouse IgG, Alexa Fluor 488 mouse IgG, Alexa Fluor 405 mouse IgG, Alexa Fluor 546 rabbit IgG, Alexa Fluor 488 rabbit IgG and Alexa Fluor 488 goat IgG antibodies were obtained from Molecular Probes (USA).

### Plasmid constructs

The N-terminally myc-tagged WT BACE1 was a kind gift from Dr. Bradley Hyman (Massachusetts General Hospital, Harvard Medical School, USA) [28]. D495R BACE1, replacement from aspartic acid at 495 to arginine, was generated from WT BACE1 by site-directed mutagenesis using the Quik Change site-directed mutagenesis kit (Agilent Technologies, USA). Primers: 5’-GCATGATGACTTTGCTCGTGACATCTCCCTGCTGA-3’ and 5’-TCAGCAGGGAGATGTCACGAGCAAAGTCATCATGC-3’. Human AP-2 α-subunit was cloned by PCR from human ovary cDNA (Gene Pool cDNA, Invitrogen) using these primers: 5’-TTTTGAATTCACCAAGATGCCGGCCGTGTCCAAG-3’ and 5’-TTTTCTCGAGAAACTGCGCTGAGAGCAATTCACA-3’. The PCR product was inserted into the reading frame of the EcoR1/Xho1 sites of pcDNA 3 with HA tag vector, established by Dr. Ryosuke Takahashi (Kyoto University, Japan). Precise cloning of all reading frames was verified by sequencing analysis.

### Immunoblotting

For SDS-PAGE, the mice brains were extracted in RIPA buffer with protease inhibitor cocktail and sufficiently homogenized on ice. Then, the samples were incubated for one night at 4°C. Cultured cells were washed twice in PBS, and scraped off. The cell pellets were suspended in TNE buffer supplemented with protease inhibitor cocktail. Each sample from mice brains or cultured cells was then centrifuged at 14,000 g for 20 minutes at 4°C, and the supernatants were collected. Protein concentration was determined using the Bradford assay. The following protocol has been described previously [29, 30]. 4–12% NuPAGE Bis-Tris gel (Life technology) was used to detect APP CTFβ. For Native-PAGE, the mice brains and cultured cells were extracted in TNE buffer with protease inhibitor cocktail, followed by 10 times sonication. Each sample from mice brains or cultured cells was then centrifuged at 14,000 g for 5 minutes at 4°C, and the supernatants were collected. Protein concentration was determined using the Bradford assay. 20 μg samples were electrophoresed on 3–12% Native PAGE Bis-Tris Protein Gels (Life technology) in anode buffer (0.1 M Tris HCl, pH 7.8), cathode buffer (0.068 M Glycine, 0.053 M Tris HCl, pH 8.9). Then, the proteins on the gel were transferred to PVDF membrane using Wet/Tank Blotting Systems (Bio-Rad) in Towbin buffer (Tris 3.0 g, Glycine 14.4 g, methanol 150 ml/ 1L). The PVDF was stained by Panceau. The following protocol was the same as that of SDS-PAGE. Signals on films were quantified with National Institutes of Health Image software (Image J).

### Immunostaining and internalization assay

APPsw/Ncad-CHO cells were harvested onto 0.1% polyethyleneimine-coated Lab-Tek II Chamber Slides (Nalge Nunc International, USA) at a density of 1.0 × 10^5^ /well and maintained in Opti-MEM containing 10% FBS for 24 hours. APPsw/Ncad-CHO cells were transfected with each constructs and 24 hours after transfection, the cells were fixed with 4% paraformaldehyde for 20 minutes. The fixed cells were blocked with PBS containing 0.2% Triton X-100 for 15 minutes and incubated with primary antibodies for 1 hour. The cells were then incubated with fluorescent-labelled second antibodies for 1 hour. To examine the internalizing process of the cell surface BACE1, an internalization assay was conducted as described previously [28]. Briefly, APPsw/Ncad-CHO cells were harvested onto 0.1% polyethyleneimine-coated Lab-Tek II Chamber Slides at a density of 1.0 × 10^5^ /well and maintained in Opti-MEM containing 10% FBS for 24 hours. The cells were transfected with each constructs. 24 hours after transfection, the cells were incubated with Opti-MEM containing an anti-myc antibody to label the surface BACE1 for 1 hour at 4°C, and the cells were washed with ice cold PBS three times. Then, the cells were returned to 37°C for indicated intervals, 0, 5 or 15 minutes. The cells were fixed with 4% paraformaldehyde for 20 minutes. The fixed cells were blocked with PBS containing 0.2% Triton X-100 for 15 minutes, and incubated with primary antibodies for 1 hour. The cells were then incubated with fluorescent-labelled second antibodies for 1 hour. All images were visually analysed using a laser confocal scanning microscope, FV10i-LIV (Olympus Corporation, Japan).

### Measurement of extracellular sAPPβ and Aβ

The levels of Swedish sAPPβ, WT sAPPβ, Aβ 40 and Aβ 42 were measured by ELISA kits specific to Swedish sAPPβ, WT sAPPβ, Aβ 40 and Aβ 42 (Immuno-Biological Laboratories Co., Ltd., Japan), according to the manufacturer’s instructions. Briefly, APPsw/Ncad-CHO cells were cultured in 3.5cm dishes at a density of 6.0 × 10^5^ cells/well. SH-SY5Y cells were cultured in 3.5cm dishes at a density of 1.2 × 10^6^ cells/well. 24 hours after incubation, the cells were transfected with the constructs. 24 hours after transfection, the cells were washed 2 times with Opti-MEM and incubated with 1 ml of Opti-MEM. 9–12 hours after replacing the culture medium, each medium was collected and centrifuged at 600 g for 5 minutes. 850 μl from the supernatant was collected for ELISA samples. The level of sAPPβ was normalized by the BACE1 level transiently overexpressed.

### Biotinylation of cell surface proteins

APPsw/Ncad-CHO cells were cultured in 10cm dishes at a density of 3.0 × 10^6^ cells/dish and 48 hours after incubation, the cells were transfected with the constructs. 24 hours after transfection, the cells were suspended in PBS containing Sulfo-NHS-LC-Biotin (Pierce Biotechnology, USA) (0.3 mg/ml) for 30 minutes at 4°C. The cells were then washed with PBS and biotinylated proteins were precipitated by 40 μl of streptoavidin agarose (Invitrogen) from equal amounts of cell lysates. Precipitated biotinylated proteins were washed with TNE buffer 4 times and re-suspended in 25 μl 2 × sample buffer. After boiling for 5 minutes and centrifuging at 14,000 g for 5 minutes, the supernatants were then subjected to immunoblotting analysis.

### Measurement of BACE1 activity

APPsw/Ncad-CHO cells were cultured in 3.5cm dishes at a density of 3.6 × 10^5^ cells/well and 24 hours after incubation, they were transfected with the constructs. 24 hours after transfection, the cells were washed 2 times by PBS, collected, and lysed by adding 100 μl of Extraction Buffer by pipetting 20 times. Then, BACE1 activity was measured by using β-secretase activity kit (Abcam) according to the manufacturer’s protocol. Fluorescence was determined using Infinite 200 PRO (Tecan Japan Co., Ltd., Japan) (excitation 345 nm, emission 505 nm).

### Statistical analysis

All values are given in means ± SE. Comparisons were performed using an unpaired Student’s t-test. For comparison of multi-parametric analysis, one-way factorial ANOVA followed by post-hoc analysis by Fisher Protected Least Significant Difference (PLSD) was used. p< 0.05 was considered to indicate a significant difference.

## Results

### HFD increased the amount of BACE1/clathrin complex in APP transgenic mice

**Fig 1:**
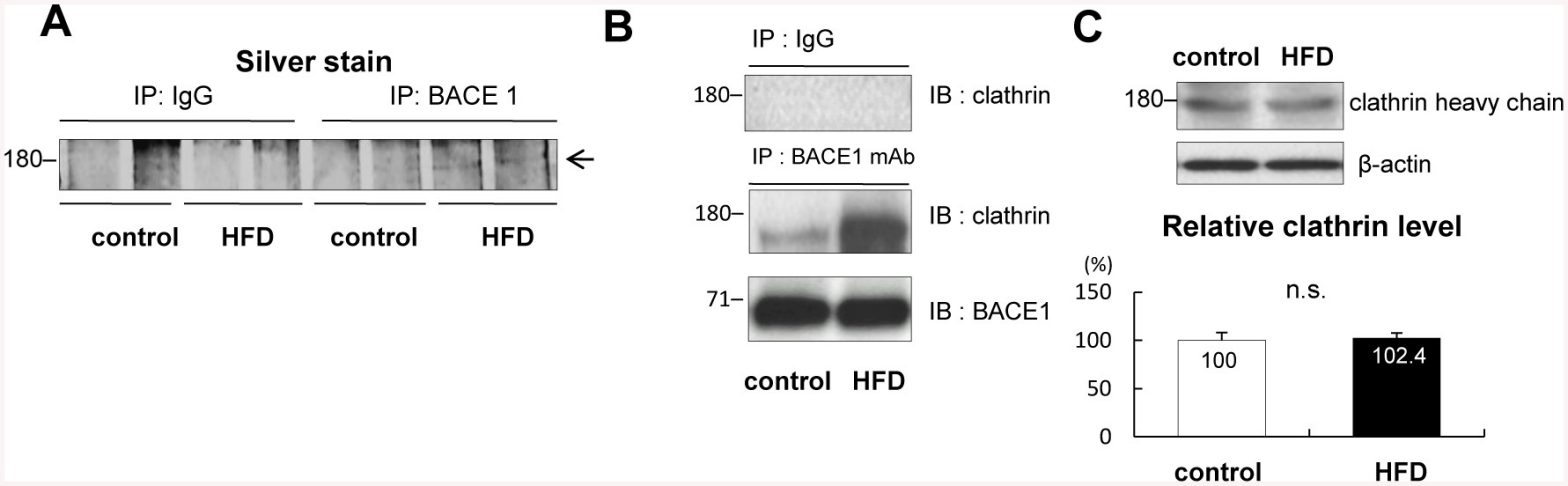
HFD promoted BACE1/clathrin complex formation in APP transgenic mice. *(A)* Equal amounts of brain samples from 7–8 months age of the control APP mice and APP-HFD mice (fed with HFD for 20 weeks) were immunoprecipitated by an anti-BACE1 antibody, and then SDS-PAGE was conducted. As a negative control, samples were immunoprecipitated by a normal mouse IgG. To visualize proteins, the gels were stained with silver nitrate. These proteomics assays were repeated twice using different four brain samples from the control APP mice as well as four samples from APP-HFD mice. The band density around 180 kDa was significantly higher in APP-HFD mice than that in the control APP mice. The protein bands were excised and subjected to in gel trypsinization, and molecular mass analysis of the tryptic peptides indicated that the band was clathrin heavy chain. (*B)* Equal amounts of brain samples from 7–8 months age of the control APP mice and APP-HFD mice (fed with HFD for 20 weeks) were immunoprecipitated by a monoclonal anti-BACE1 antibody (2^nd^ row) and then they were immunoblotted by an anti-clathrin antibody or by an anti-BACE1 antibody. As a negative control, samples were immunoprecipitated by a normal mouse IgG (top row). These immunoprecipitation assays were repeated twice using different four brain samples from the control APP mice as well as four samples from APP-HFD mice. HFD promoted the BACE1/clathrin complex formation in APP transgenic mice. *(C*) Immunoblotting analysis using an anti-clathrin antibody. Statistical analysis is shown in the bottom panel. The band density of the clathrin heavy chain was normalized by that of β-actin. The band density of the control APP mice was regarded as 100% and that of APP-HFD mice was relatively indicated. The band density of clathrin in APP-HFD mice (fed with HFD for 20 weeks) was the same as that in the control APP mice. n.s. indicates that there was no statistical significance.

In a previous study, we observed that APP-CTFβ had accumulated in the brains of APP transgenic mice fed with HFD for 20 weeks (APP-HFD mice), compared with that in the control APP mice. Moreover, HFD did not affect on the levels of APP or BACE1, suggesting that HFD may specifically strengthen BACE1 activity, thus promoting of the cleavage of APP by BACE1 [20]. To demonstrate the mechanism through which HFD promotes BACE1-mediated APP cleavage, we set out to identify the proteins that associate with BACE1 in mice fed with HFD. For this, lysates from the brains of the control APP mice as well as those from APP-HFD mice were immunoprecipitated with an anti-BACE1 antibody, and subjected to SDS-PAGE. The separated proteins were visualized by silver staining, demonstrating a band density of around 180 kDa in the samples of the APP-HFD mice stronger than that of the control APP mice (Fig 1A). The protein band was identified by mass spectrometry as clathrin heavy chain. Clathrin heavy chain is a component of clathrin vesicles; they form on the cell membrane and are associated with internalization of receptors as well as ligands [31]. To confirm the complex formation of BACE1 with clathrin, fresh mice brain lysates were immunoprecipitated with an anti-BACE1 antibody. Immunoblotting analysis indicated that the amount of BACE1/clathrin complex in APP-HFD mice was greater than that in the control APP mice (Fig 1B). Also, the level of clathrin in APP-HFD mice was not different from that in the control APP mice (Fig 1C). These results indicated that HFD might promote BACE1/clathrin complex formation without changing the level of clathrin in APP transgenic mice.

### AP-2 mediated the BACE1/clathrin complex formation in APP transgenic mice

**Fig 2:**
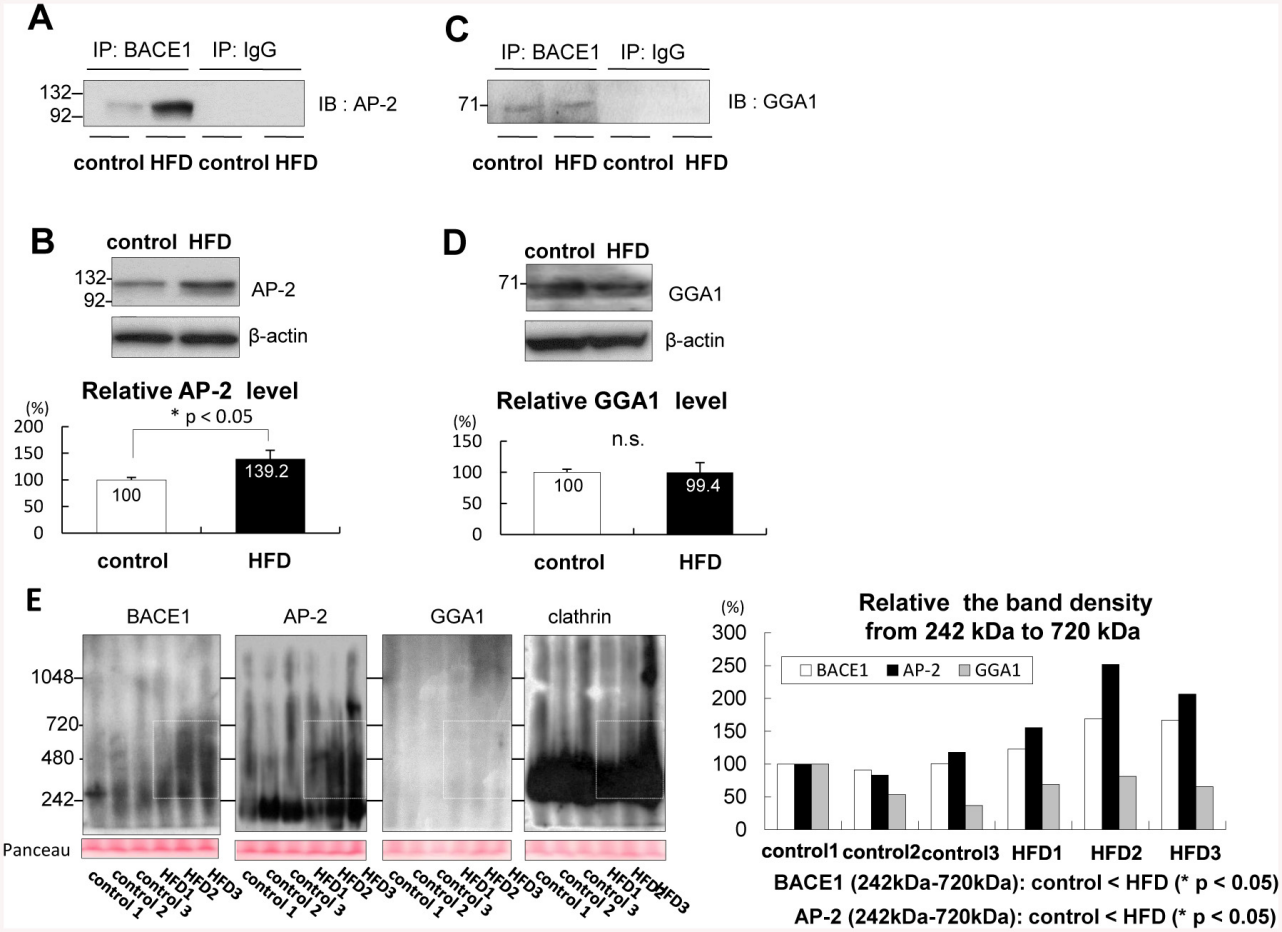
AP-2 mediated BACE1/clathrin complex in APP transgenic mice. *(A)* Equal amounts of brain samples from 7–8 months age of the control APP mice and APP-HFD mice (fed with HFD for 20 weeks) were immunoprecipitated by an anti-BACE1 antibody, and then immunoblotted by an anti-AP-2α antibody (1^st^- 2^nd^ lane). As a negative control, samples were immunoprecipitated by a normal mouse IgG (3^rd^- 4^th^ lane). These immunoprecipitation assays were repeated twice using different two brain samples from the control APP mice as well as two samples from APP-HFD mice. HFD promoted BACE1/AP-2/clathrin complex formation. *(B*) Immunoblotting analysis using an anti-AP-2α antibody. Statistical analysis is shown in the bottom panel. The band density of AP-2 was normalized by that of β-actin. The band density of the control APP mice was regarded as 100% and that of APP-HFD mice was relatively indicated. The band density of AP-2 in APP-HFD mice (fed with HFD for 20 weeks) was significantly higher than that in the control APP mice (n = 4, p = 0.041). * indicates p < 0.05. (*C)* Equal amounts of brain samples from 7–8 months age of the control APP mice and APP-HFD mice (fed with HFD for 20 weeks) were immunoprecipitated by an anti-BACE1 antibody, and then immunoblotted by an anti-GGA1 antibody (1^st^- 2^nd^ lane). As a negative control, samples were immunoprecipitated by a normal mouse IgG (3^rd^- 4^th^ lane). BACE1 and GGA1 formed complex in APP-HFD mice the same as in the control APP mice. *(D*) Immunoblotting analysis using an anti-GGA1 antibody. Statistical analysis is shown in the bottom panel. The band density of GGA1 was normalized by that of β-actin. The band density of the control APP mice was regarded as 100% and that of APP-HFD mice was relatively indicated. The band density of GGA1 in APP-HFD mice (fed with HFD for 20 weeks) was the same as that in the control APP mice (n = 3). n.s. indicates that there was no statistical significance. (*E*) Immunoblotting analysis in the native condition. Equal amounts of brain samples from 7–8 months age of the control APP mice (n = 3) and APP-HFD mice (fed with HFD for 20 weeks) (n = 3) were separated on Native-PAGE gel, followed by detection using anti-BACE1 (1^st^ panel), anti-AP-2α (2^nd^ panel), anti-GGA1 (3^rd^ panel) and anti-clathrin antibodies (4^th^ panel). The band density in the area from 242 kDa to 720 kDa (white rectangle) was normalized by the density of Panceau, and statistical analysis is shown in the right panel. The band density of the control APP mice (sample 1) was regarded as 100% and that of other mice was relatively indicated. The band density of BACE1 in the area from 242 kDa to 720 kDa did not match that of GGA1 but clearly matched that of AP-2 in each sample. The average band densities of BACE1 (p = 0.038) as well as AP-2 (p = 0.041) in the area from 242 kDa to 720 kDa in APP-HFD mice were higher than those in the control APP mice.

It has been previously reported that either the AP-2 complex (α-β2-μ2-σ2) [32, 33] or Golgi-localized, gamma adaptin ear-containing, ARF-binding protein 1 (GGA1) [34] acts to mediate the BACE1/clathrin complex formation. To reveal the mediator of BACE1/clathrin complex formation in APP transgenic mice, brain lysates were immunoprecipitated with an anti-BACE1 antibody. Immunoblotting analysis using an anti-AP-2α antibody indicated that BACE1/AP-2 interaction was enhanced in APP-HFD mice relative to that in the control APP mice (Fig 2A). To study the mechanistic link in these results, we examined the protein level of AP-2, which was significantly higher in APP-HFD mice than that in the control APP mice (Fig 2B). On the other hand, immunoblotting analysis using an anti-GGA1 antibody indicated that the amount of BACE1/GGA1 complex in APP-HFD mice was not different from that in the control APP mice (Fig 2C). Moreover, the expression level of GGA1 in APP-HFD mice was the same as that in the control APP mice (Fig 2D). In order to confirm the results from immunoprecipitation assays, we also conducted Native-PAGE using anti-BACE1, anti-AP-2α, anti-GGA1 and anti-clathrin antibodies. Immunoblotting analysis in the native condition showed more BACE1-associating aggregates in the area between 242 kDa and 720 kDa in APP-HFD mice compared to in the control APP mice. Importantly, these aggregates were not detected by an anti-GGA1 antibody but clearly detected by an anti-AP-2α antibody. Many clathrin-associating aggregates were also observed in this area, both in APP-HFD mice and in the control APP mice (Fig 2E). These results indicated that AP-2 might mediate the BACE1/clathrin complex formation in response to HFD in APP transgenic mice.

### D495R BACE1 decreased the sAPPβ level

**Fig 3:**
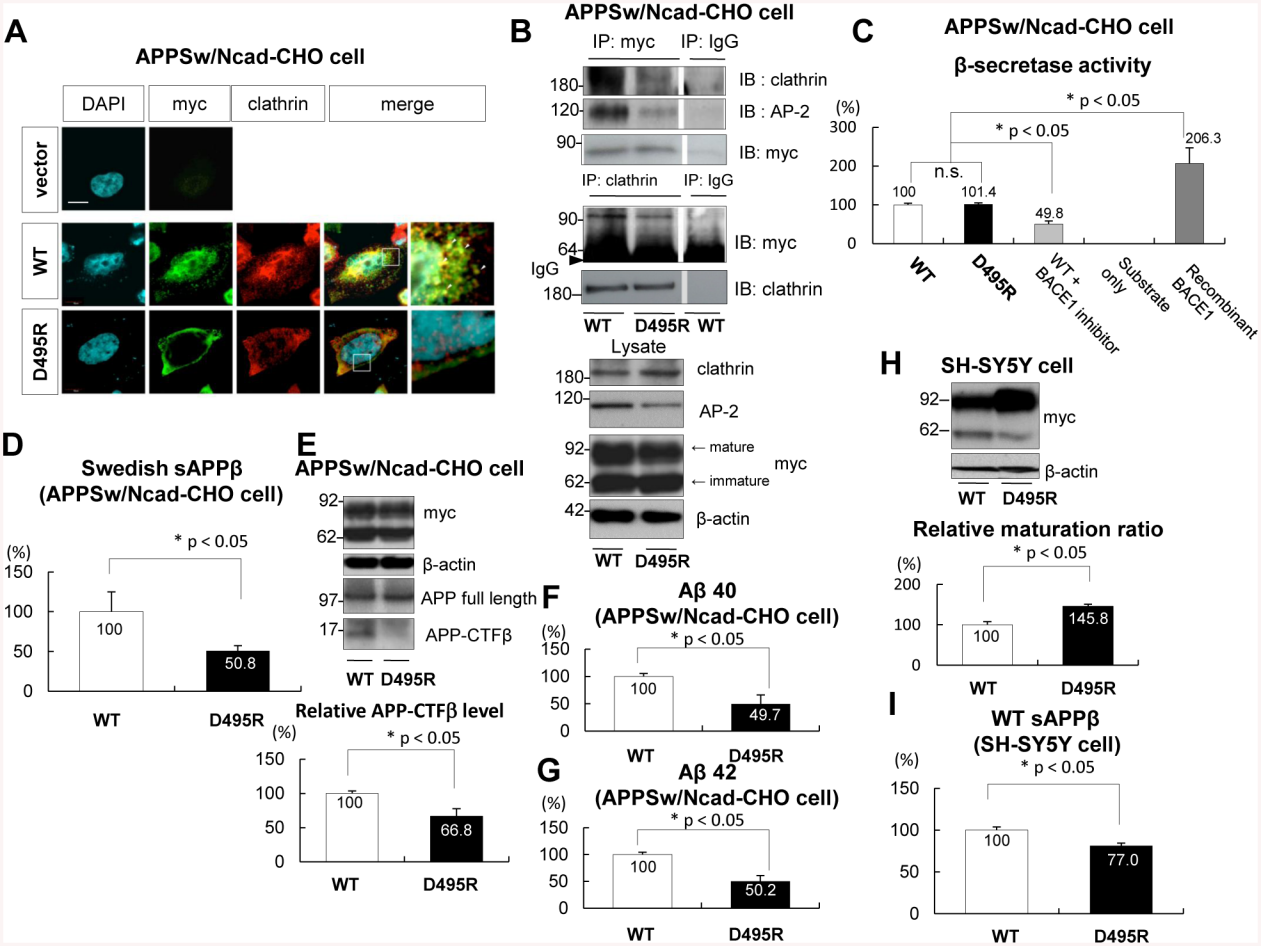
D495R BACE1 inhibited the cleavage of APP in Swedish APP overexpressing CHO cells and in SH-SY5Y cells. (A) Immunostaining analysis to examine the localizations of BACE1 and clathrin using an anti-myc antibody (2^nd^ lane) and an anti-clathrin antibody (3^rd^ lane). APPsw/Ncad-CHO cells were transfected either with WT BACE1 (middle row) or with D495R BACE1 (bottom row). Myc tag was linked to these BACE1 constructs. As a negative control, the cells were overexpressing empty vector (top row). The nucleus of the cell was labelled by DAPI (1^st^ lane). High-magnification images are shown in the 5^th^ lane. WT BACE1 was co-localized with clathrin in the cytoplasmic vesicles (arrowhead). On the other hand, D495R BACE1 was localized on the plasma membrane and hardly co-localized with clathrin. Scale bar indicates 10 μm. (B) APPsw/Ncad-CHO cells were transfected either with WT BACE1 (1^st^ and 3^rd^ lanes) or with D495R BACE1 (2^nd^ lane). Myc tag was linked to these BACE1 constructs. Equal amounts of cell lysates were immunoprecipitated by an anti-myc antibody, and then immunoblotted by an anti-clathrin antibody (upper panel, 1^st^ row), an anti-AP-2α antibody (2^nd^ row) and an anti-myc antibody (3^rd^ row). As a negative control, samples were immunoprecipitated by a normal rabbit IgG (3^rd^ lane). To confirm these results, samples were immunoprecipitated by an anti-clathrin antibody and immunoblotted by an anti-myc antibody (4^th^ row) or by an anti-clathrin antibody (5^th^ row). The expression levels of each protein in cell lysates are shown in the bottom panel. The level of BACE1/AP-2/clathrin complex was decreased in D495R BACE1 expressing cells compared with in WT BACE1 cells. (C) APPsw/Ncad-CHO cells were transfected either with WT BACE1 (1^st^, 3^rd^ and 4^th^ lanes) or with D495R BACE1 (2^nd^ lane). These cells were lysed, and β-secretase activity in the cell lysates was measured by the β-secretase activity assay kit. All values were subtracted from the background readings obtained from only substrate (without secretase) (4^th^ lane). As a negative control, the CHO cells transfected WT BACE1 were treated with the BACE1 inhibitor which was included in the kit (3^rd^ lane). As a positive control, the substrate was mixed with the recombinant active BACE1which was also included in the kit (5^th^ lane). In vitro condition, the BACE1 activity in D495R BACE1 transfected cell was not different from that in WT BACE1 (n = 12, F _(4,31)_ = 32.48). n.s. indicates that there was no statistical significance. (D) APPsw/Ncad-CHO cells were transfected either with WT BACE1 (1^st^ lane) or with D495R BACE1 (2^nd^ lane). The levels of extracellular Swedish sAPPβ were determined by the Swedish sAPPβ specific ELISA kit. The levels of sAPPβ were normalized by the levels of BACE1 (mature plus immature forms) transiently overexpressed. The average level of sAPPβ in WT BACE1-transfected cells was regarded as 100% and that in D495R BACE1-transfected cells was relatively indicated. The level of Swedish sAPPβ in D495R BACE1-transfected cell was significantly smaller than that in WT BACE1 (n = 12, p = 0.034). * indicated p < 0.05. (E) Immunoblotting analysis using an anti-Aβ 6E10 antibody which only detects APP-CTFβ. APPsw/Ncad-CHO cells were transfected either with WT BACE1 (1^st^ lane) or with D495R BACE1 (2^nd^ lane). The maturation of WT BACE1 was not different with that of D495R BACE1. Although the band density of APP full length was not different, the band density of APP-CTFβ in D495R-transfected cells was significantly lower than that in WT BACE1 (n = 5, p = 0.009). * indicated p < 0.05. (F) APPsw/Ncad-CHO cells were transfected either with WT BACE1 (1^st^ lane) or with D495R BACE1 (2^nd^ lane). The levels of extracellular Aβ 40 were determined by the Aβ 40 specific ELISA kit. The average level of Aβ 40 in WT BACE1-transfected cells was regarded as 100% and that in D495R BACE1-transfected cells was relatively indicated. The level of Swedish Aβ 40 in D495R BACE1-transfected cell was significantly smaller than that in WT BACE1 (n = 12, p = 0.037). * indicated p < 0.05. (G) APPsw/Ncad-CHO cells were transfected either with WT BACE1 (1^st^ lane) or with D495R BACE1 (2^nd^ lane). The levels of extracellular Aβ 42 were determined by the Aβ 42 specific ELISA kit. The average level of Aβ 42 in WT BACE1-transfected cells was regarded as 100% and that in D495R BACE1-transfected cells was relatively indicated. The level of Swedish Aβ 42 in D495R BACE1-transfected cell was significantly smaller than that in WT BACE1 (n = 12, p = 0.003). * indicated p < 0.05. (H) Immunoblotting analysis using an anti-myc antibody. SH-SY5Y cells were transfected either with WT BACE1 (1^st^ lane) or with D495R BACE1 (2^nd^ lane). The maturation was analysed by the relative ration of the mature form of BACE1/the immature form of BACE1. The maturation of D495R BACE1 was significantly promoted compared to that of WT BACE1 (n = 6, p = 0.0001). * indicates p < 0.05. (I) SH-SY5Y cells were transfected either with WT BACE1 (1^st^ lane) or with D495R BACE1 (2^nd^ lane). The levels of extracellular WT sAPPβ were determined by the WT sAPPβ specific ELISA kit. The levels of sAPPβ were normalized by the levels of BACE1 (mature form) transiently overexpressed. The average level of sAPPβ in WT BACE1-transfected cells was regarded as 100% and that in D495R BACE1-transfected cells is relatively indicated. The level of WT sAPPβ in D495R BACE1-transfected cell was significantly smaller than that in WT BACE1 (n = 15, p = 0.0007). * indicates p < 0.05.

A recent report showed that AP-2 interacts with BACE1 at the first aspartate (D 495) residue of the DDISLL sequence, while the artificial mutant D495R BACE1 inhibits the BACE1/AP-2 interaction [32]. First, to examine the effect of BACE1/AP-2/clathrin complex formation on APP metabolism, we inhibited the trimeric complex formation by overexpressing D495R BACE1 in Swedish APP overexpressing CHO cells. Immunostaining analysis indicated that D495R BACE1 mainly localized at the plasma membrane. On the other hand, some WT BACE1 localized at clathrin positive vesicles in the cytosolic region (Fig 3A). An immunoprecipitation assay confirmed that D495R mutation inhibited BACE1 interaction with AP-2 as well as with clathrin (Fig 3B). An *in vitro* β-secretase activity assay indicated that the enzymatic activity of D495R BACE1 in the artificial reaction buffer was not different from that of WT BACE1, indicating that D495R mutation did not work as a dominant negative mutation on BACE1 (Fig 3C). Importantly, ELISA indicated that the level of Swedish sAPPβ in D495R BACE1-transfected cells was significantly lower than that in WT BACE1-transfected cells (Fig 3D), which was confirmed by examination of APP-CTFβ level by immunoblotting (Fig 3E). Furthermore, the levels of extracellular Aβ 40 and Aβ 42 in D495R BACE1-transfected cells were also significantly lower than those in WT BACE1-transfected cells (Fig 3F and 3G). In order to examine whether the results from Swedish APP overexpressing CHO cells were relevant to those of WT APP expressing neuronal cells, we investigated the role of BACE1/AP-2/clathrin complex in SH-SY5Y cells. Interestingly, although it was not observed in the APP overexpressing CHO cells (Fig 3E), the maturation of D495R BACE1 was significantly promoted compared to that of WT BACE1 in SH-SY5Y cells (Fig 3H). Since the maturation of BACE1 is tightly associated with its proteolytic activity [35, 36], the levels of WT sAPPβ were normalized by those of mature BACE1. Importantly, ELISA indicated that the level of WT sAPPβ in D495R BACE1-transfected SH-SY5Y cells was significantly lower than that in WT BACE1-transfected cells (Fig 3I). These results indicated that inhibition of BACE1/AP-2/clathrin complex formation might down-regulate the β-site cleavage of APP, followed by decrease in levels of sAPPβ, APP-CTFβ and Aβ.

### Overexpression of AP-2 increased the sAPPβ level

**Fig 4:**
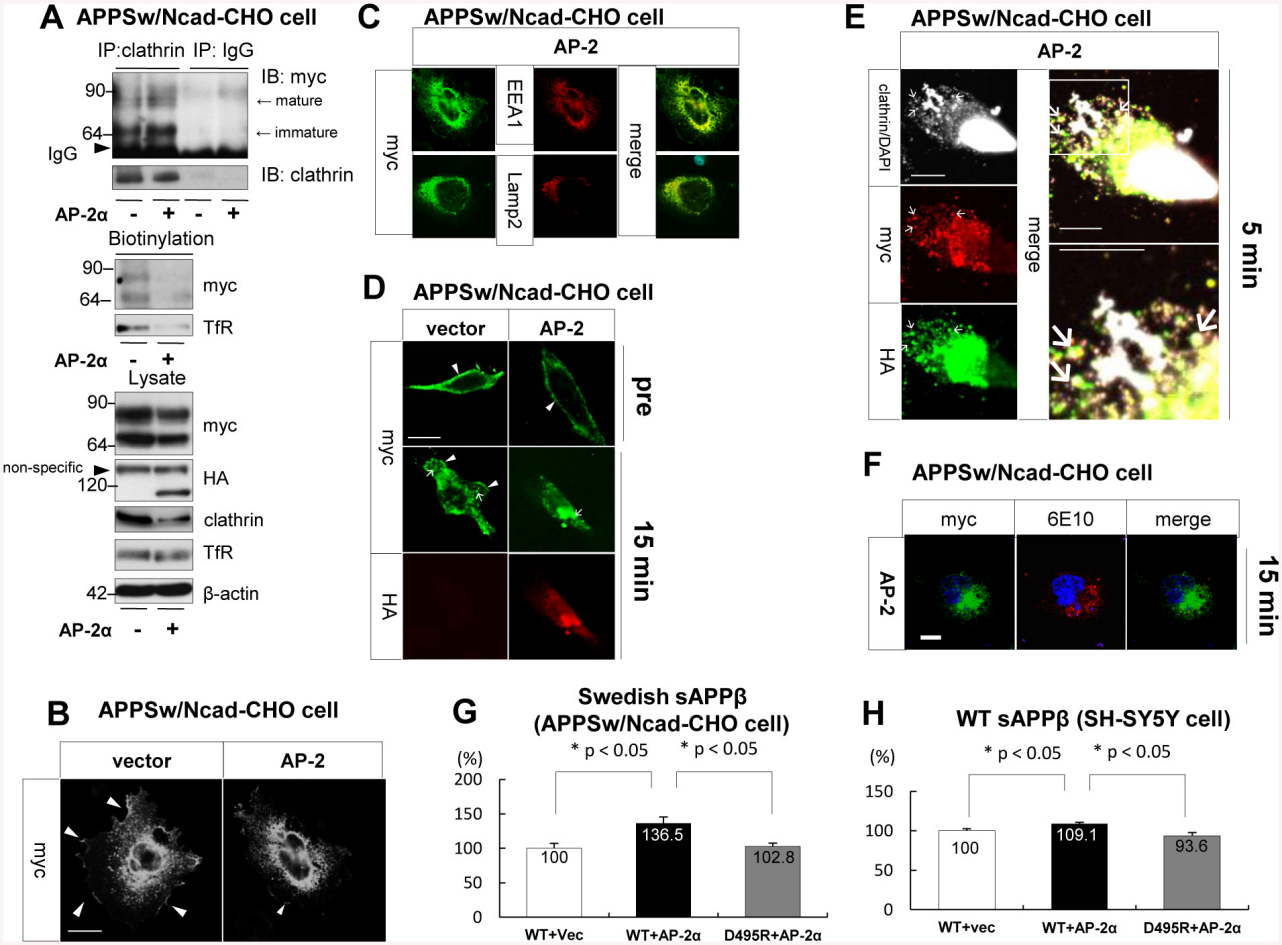
Overexpression of AP-2 promoted the cleavage of APP by BACE1 in Swedish APP overexpressing CHO cells and in SH-SY5Y cells. (A) APPsw/Ncad-CHO cells were transfected either with WT BACE1 and empty vector (1^st^ and 3^rd^ lanes) or with WT BACE1 and AP-2α (2^nd^ and 4^th^ lanes). Myc tag was linked to WT BACE1 and HA tag was linked to AP-2α. Equal amounts of cell lysate were immunoprecipitated by an anti-clathrin antibody, and then immunoblotted by an anti-myc antibody (upper panel, 1^st^ and 2^nd^ lanes). As a negative control, samples were immunoprecipitated by a normal mouse IgG (3^rd^ and 4^th^ lanes). The CHO cells overexpressing either with WT BACE1 and empty vector or with WT BACE1 and AP-2α were biotinylated, and cell surface level of BACE1 was examined by an anti-myc antibody (middle panel, 1^st^ row). As a positive control, cell surface level of TfR was examined (middle panel, 2^nd^ row). The expression levels of each protein in cell lysates are shown in the bottom panel. Overexpression of AP-2 increased the amount of BACE1/clathrin complex, and decreased the cell surface level of BACE1. (B) Immunostaining analysis to examine the localizations of BACE1 using an anti-myc antibody under AP-2 overexpressing conditions. APPsw/Ncad-CHO cells were transfected with WT BACE1 and AP-2α (right panel). As a negative control, the cells were overexpressing WT BACE1 and empty vector (left panel). Myc tag was linked to WT BACE1. WT BACE1 was localized on the cell surface (arrowhead) as well as in the intracellular compartments in a negative control cells. On the other hand, WT BACE1was hardly localized on the plasma membrane and mainly localized in the intracellular compartments under AP-2 overexpressing conditions. Scale bar indicates 10 μm. (C) Immunostaining analysis to examine the detailed localizations of BACE1 using anti-EEA1 and anti-Lamp2 antibodies under AP-2 overexpressing conditions. Myc tag was linked to WT BACE1. EEA1 was used as an endosome marker and Lamp2 was a lysosome marker. Under the AP-2 overexpressing condition, WT BACE1 was localized in the endosome and in the lysosome. (D) An internalization assay to examine the localization change of cell surface BACE1. APPsw/Ncad-CHO cells were transfected with WT BACE1 and empty vector (left panel) or with WT BACE1 and AP-2α (right panel). The cell surface BACE1 was labeled by an anti-myc antibody, followed by examination of its localization before (upper row) and 15 minutes after incubation (middle row). The expression of AP-2α was checked by an anti-HA antibody (bottom row). 15 minutes after incubation, the BACE1 labelled by an anti-myc antibody was observed on the cell membrane (arrowhead) and at the intracellular vesicles (arrow) in empty vector-transfected cells. On the other hand, the BACE1 was clearly accumulated in the intracellular compartment (arrow) and it was hardly observed on the cell membrane in the AP-2 overexpresing cells. (E) Triple immunostaining analysis for detecting BACE1/AP-2/clathrin complex in the internalization assay. APPsw/Ncad-CHO cells were transfected with WT BACE1 and AP-2α. Cell surface BACE1 was labelled by an anti-myc antibody. Clathrin and AP-2 were stained with anti-clathrin and anti-HA antibodies respectively. BACE1/AP-2/clathrin complex formed during the internalization of cell surface BACE1 (5 minutes after incubation) is indicated arrow. Right upper panel is a merged image and right bottom panel is high magnification of the image contained in the rectangle in the upper panel. Scale bar indicates 10 μm. (F) Immunostaining analysis to examine whether the internalized BACE1 co-localized with APP under AP-2 overexpressing conditions. Myc tag was linked to WT BACE1. The cell surface BACE1 was labelled by an anti-myc antibody, followed by incubating for 15 minutes. APP was stained by 6E10 antibody. 15 minutes after incubation, the BACE1 labelled by an anti-myc antibody was not clearly co-localized with APP. On the other hand, APP remarkably surrounded the compartment in which the BACE1 was accumulated. Scale bar indicates 10 μm. (G) APPsw/Ncad-CHO cells were transfected with WT BACE1 and empty vector (1^st^ lane), with WT BACE1 and AP-2α (2^nd^ lane) or with D495R BACE1 and AP-2α (3^rd^ lane). The levels of extracellular Swedish sAPPβ were determined by the Swedish sAPPβ specific ELISA kit. The levels of sAPPβ were normalized by the levels of BACE1 (mature form plus immature) transiently overexpressed. The average level of sAPPβ in WT BACE1 with empty vector-transfected cells was regarded as 100% and that in other cells was relatively indicated. The level of Swedish sAPPβ in WT BACE1 and AP-2α-transfected cell was significantly higher than that in WT BACE1 and empty vector (n = 12, F _(2,33)_ = 7.86, p = 0.001). On the other hand, the level in D495R BACE1 and AP-2α-transfected cell was lower than that in WT BACE1 and AP-2α-transfected cell (p = 0.002). * indicates p < 0.05. (H) SH-SY5Y cells were transfected with WT BACE1 and empty vector (1^st^ lane), with WT BACE1 and AP-2α (2^nd^ lane) or with D495R BACE1 and AP-2α (3^rd^ lane). The levels of extracellular WT sAPPβ were determined by the WT sAPPβ specific ELISA kit. The levels of sAPPβ were normalized by the levels of BACE1 (mature form) transiently overexpressed. The average level of sAPPβ in WT BACE1 with empty vector-transfected cells was regarded as 100% and that in other cells was relatively indicated. The level of WT sAPPβ in WT BACE1 and AP-2α-transfected cell was significantly higher than that in WT BACE1 and empty vector (n = 14, F _(2,39)_ = 6.34, p = 0.045). On the other hand, the level in D495R BACE1 and AP-2α-transfected cell was lower than that in WT BACE1 and AP-2α-transfected cell (p = 0.001). * indicates p < 0.05.

Next, we promoted the formation of the BACE1/AP-2/clathrin complex by AP-2 overexpression in Swedish APP overexpressing CHO cells. An immunoprecipitation assay using an anti-clathrin antibody indicated that overexpression of AP-2 increased the amount of BACE1/clathrin complex (Fig 4A). We also conducted a biotinylation assay, demonstrating that overexpression of AP-2 decreased the cell surface level of BACE1 (Fig 4A). Similarly, the overexpression of AP-2 decreased the cell surface level of Transferrin receptor (TfR), which is endocytosed by AP-2/clathrin vesicle [37]. Immunostaining analysis also showed that AP-2 overexpression reduced the cell surface level of BACE1 (Fig 4B). BACE1 was co-localized with Early Endosome Antigen 1 (EEA1); an endosome marker, and also with Lysosome-associated membrane protein 2 (Lamp2); a lysosome marker under AP-2 overexpressing conditions (Fig 4C). Moreover, in order to observe the internalization of cell surface BACE1, we conducted an internalization assay by labeling cell surface BACE1 with an anti-myc antibody. In the empty vector-transfected cells, anti-myc antibody-labeled BACE1 was observed both on the cell membrane and at the intracellular vesicles 15 minutes after incubation. On the other hand, in the AP-2 overexpressing cells, anti-myc antibody-labeled BACE1 was clearly accumulated in the intracellular compartment while it was hardly observed on the cell membrane (Fig 4D). During the internalizing process (5 minutes after incubation), BACE1 formed a complex with AP-2 and clathrin (Fig 4E). Interestingly, anti-myc labeled BACE1 did not completely co-localize with APP 15 minutes after incubation, but APP remarkably localized around the region BACE1 was accumulated (Fig 4F). ELISA indicated that overexpression of AP-2 significantly increased sAPPβ level in Swedish APP overexpressing CHO cells. On the other hand, D495R BACE1 suppressed the level of sAPPβ under AP-2 overexpressing conditions (Fig 4G). As well as in the APP overexpressing CHO cells, overexpression of AP-2 significantly increased the sAPPβ level in SH-SY5Y cells, and it was rescued by the expression of D495R BACE1 (Fig 4H). These results indicated that enhancement of the BACE1/AP-2/clathrin complex formation might accelerate the internalization of cell surface BACE1, followed by promotion of the APP cleavage by BACE1.

## Discussion

A recent population-based twin study reported that mid-life obesity increases the risk of late-life AD [38]. Moreover, a Japanese cohort Hisayama study indicated that individuals with type 2 diabetes develop deposits of Aβ [39]. As such, obesity and type 2 diabetes are highlighted as risk factors of AD. There are many experimental studies showing that HFD exacerbates Aβ deposition in several strains of APP transgenic mice [16–20]. As a mechanism, it was reported that HFD regulates γ-secretase activity [17]. In our previous report, it was stated that HFD increases the level of APP-CTFβ without promoting a change in the level of BACE1. Therefore, we hypothesized that HFD may strengthen the activity of BACE1, followed by promotion of the cleavage of APP [20]. The present study aims to understand how HFD enhances the cleavage of APP by BACE1. Here we demonstrate that HFD increased the amount of BACE1/AP-2/clathrin complex in APP transgenic mice (Figs 1 and 2). In both Swedish APP overexpressing CHO cells and SH-SY5Y cells, promotion of the trimeric complex formation increased the sAPPβ level (Fig 4). On the other hand, inhibition of the complex formation decreased the level of sAPPβ (Fig 3). Therefore, we concluded that HFD might increase the amount of BACE1/AP-2/clathrin complex, followed by enhancement of β-site cleavage of APP in APP transgenic mice.

In APP transgenic mice, HFD did not change the level of clathrin (Fig 1) or BACE1 [20]. However, HFD increased the level of AP-2, and drastically enhanced the interaction of BACE1 with AP-2 (Fig 2). In Swedish APP overexpressing CHO cells, overexpression of AP-2 increased the amount of the BACE1/AP-2/clathrin complex (Fig 4). Therefore, we speculated that HFD-induced increase of AP-2 level was the trigger for the BACE1/AP-2/clathrin complex formation in APP transgenic mice. Some reports showed that APP is cleaved by BACE1 in the trans-Golgi network [40, 41] or on the cell surface [42]. However, other reports claimed that the cleavage occurs in the endosomes [28, 43, 44]. In the present study, due to the lack of interaction with AP-2, D495R BACE1 mainly localized at the cell surface (Fig 3) and AP-2 overexpression reduced the cell surface level of BACE1 in the APP overexpressing CHO cells (Fig 4). Moreover, the internalization of BACE1 was clearly promoted under AP-2 overexpressing conditions (Fig 4). Although our present study could not clearly identify the compartment in which BACE1 primarily cleaves APP, these results suggested that the BACE1/AP-2/clathrin complex formation might lead to transportation of BACE1 from the cell surface to the intracellular compartments, which might promote the cleavage of APP by BACE1. In this sense, the AP-2/clathrin-induced endocytosis of BACE1 might be a key step of the HFD-promoted APP cleavage by BACE1 in APP transgenic mice.

Consistent with our results, a paper suggesting that knockdown of dynamin 1, which inhibits the clathrin-mediated endocytosis, down-regulates BACE1 internalization from the cell surface, and inhibits the cleavage of APP by BACE1 [45]. Dynamin 1 and AP-2 are differently involved in the clathrin-mediated endocytosis. Dynamin 1 principally undergoes the fission of newly formed clathrin-coated vesicle from the cell membrane. On the other hand, AP-2 links clathrin to molecules (i.e. BACE1) in the vesicles. Our data showed that the D495R mutation on BACE1, which inhibits interaction with AP-2, clearly increased the amount of cell surface BACE1 (Fig 3), indicating that AP-2 and dynamin 1 may independently regulate BACE1 trafficking as well as the cleavage of APP. Moreover, HFD did not affect on the expression level of dynamin 1 in APP transgenic mice (data not shown). Therefore, we proposed that HFD may not affect on dynamin 1-regulated BACE1 trafficking. Another study also demonstrated that the AP-2/clathrin-mediated BACE1 internalization enhances the cleavage of APP by BACE1. As a mechanism, the internalized BACE1 may cleave the internalized APP in the early endosome, or it may cleave the newly synthesised APP in the trans-Golgi network [33]. There is, however, a report inconsistent with ours, stating that the AP-2/clathrin-mediated BACE1 endocytosis does not affect on the APP cleavage [32]. Although the reason for the discrepancy between their results and ours is unclear, we speculate that it might at least be due to differences in experimental design. In our study, we used CHO cells stably overexpressing Swedish APP or native SH-SY5Y cells, whereas their study utilized neuroglioma-derived H4 cells or rat cortical neurons transiently overexpressing WT APP with artificial mutations inhibiting the cleavage by α-secretase and caspase. As shown in Fig 3, although WT BACE1 and D495R BACE1 were matured in the same way in CHO cells, the maturation of D495R BACE1 was significantly promoted compared to that of WT BACE1 in SH-SY5Y cells. We identified a significant decrease in the level of sAPPβ by normalizing its level with the “mature BACE1” level (Fig 3), but it was not identified when we normalized sAPPβ level with the “total (immature + mature) BACE1” level in SH-SY5Y cells (WT BACE1 100% ± 3.83 vs. D495R BACE1 98.1% ± 4.27, data not shown). Therefore, the difference of BACE1 maturation among cell types might lead to this discrepancy. In addition, the decreased level of sAPPβ by D495R BACE1 in the APP overexpressing CHO cells was greater than that in SH-SY5Y cells (Fig 3). Since Swedish APP generates a significantly larger increase in Aβ production in the endocytic compartment [28, 46], the different property in APP might also be the reason for the discrepancy.

**Fig 5:**
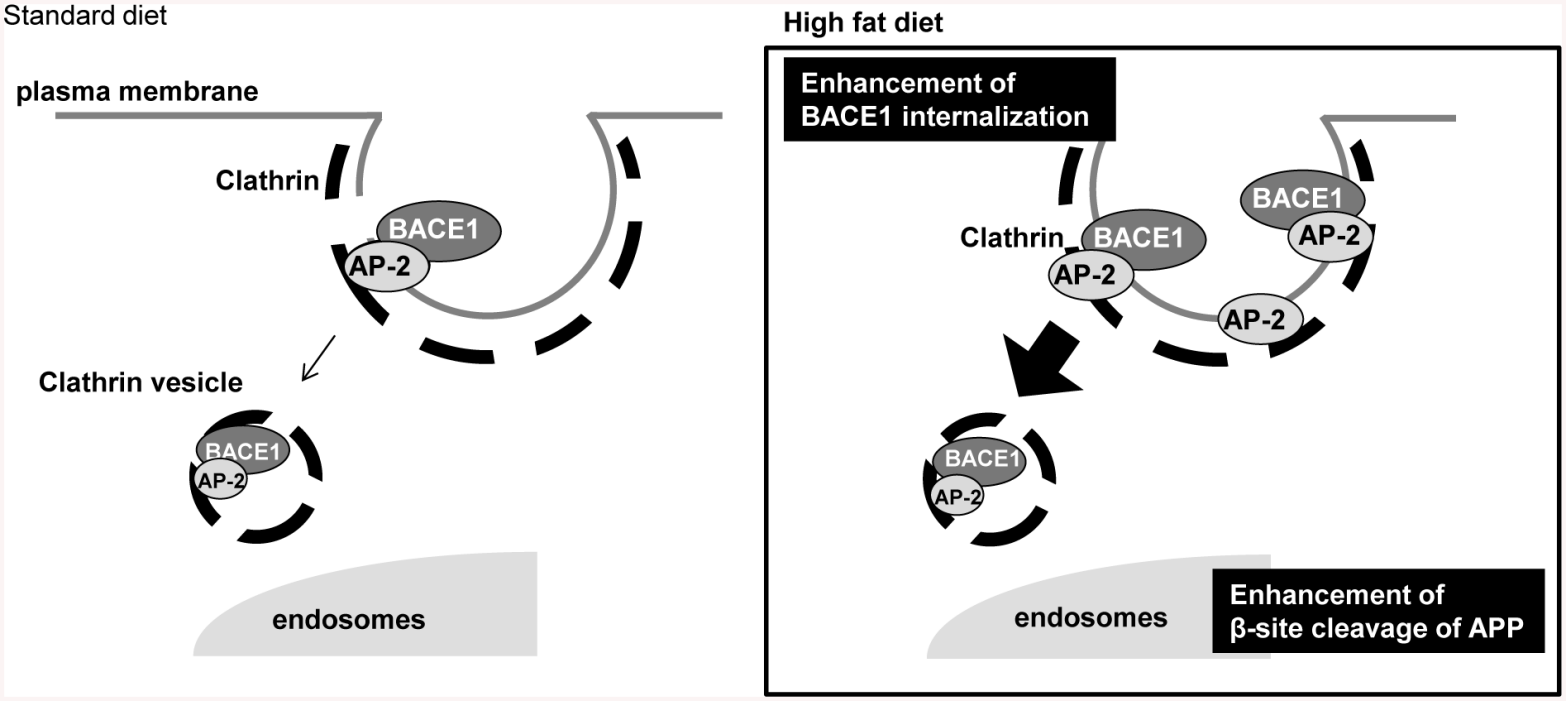
Schematic presentation of events under HFD conditions. HFD promotes the BACE1/AP-2/clathrin complex formation by increasing AP-2 levels, which accelerates the trafficking of BACE1 from the cell surface to intracellular compartments. The change in BACE1 trafficking enhances β-site APP cleavage in APP transgenic mice.

There is a limitation in the present study. We could not determine whether or not HFD had an effect on APP trafficking. Due to difficulties in labelling the cell surface APP by two different antibodies; 6E10 and 22C11 which are against N-terminus of APP (data not shown), we could not make clear the effect of AP-2 on APP trafficking. A previous paper showed that AP-2 interacts with APP on its C-terminal region [47]. Also, AP-2 knockdown clearly increases the expression level of APP [32]. These reports are suggesting that AP-2 can modulate not only BACE1 but also APP trafficking. Nevertheless, considering that obesity and type 2 diabetes are risk factors of “sporadic” AD, we should examine the effect of the BACE1/AP-2/clathrin complex on the pathology of sporadic AD in future studies. We identified the trimeric complex in the sporadic human AD brain by immunoprecipitation analysis (unpublished observation). We demonstrated for the first time a detailed mechanism of how HFD promotes APP processing by regulating BACE1 trafficking. As displayed in Fig 5, we demonstrated that HFD promoted the BACE1/AP-2/clathrin complex formation, followed by transportation of BACE1 from the cell surface to intracellular compartments. These events promoted the BACE1-mediated APP cleavage in APP transgenic mice. So far, it is widely considered that HFD interferes with insulin signalling and/or diabetic conditions, which may affect neuronal functions. We propose that HFD may regulate BACE1 function, which is associated with the exacerbation of Aβ pathology. Thus, a molecular link between dietary condition and Aβ pathology is important for the prevention of AD.
